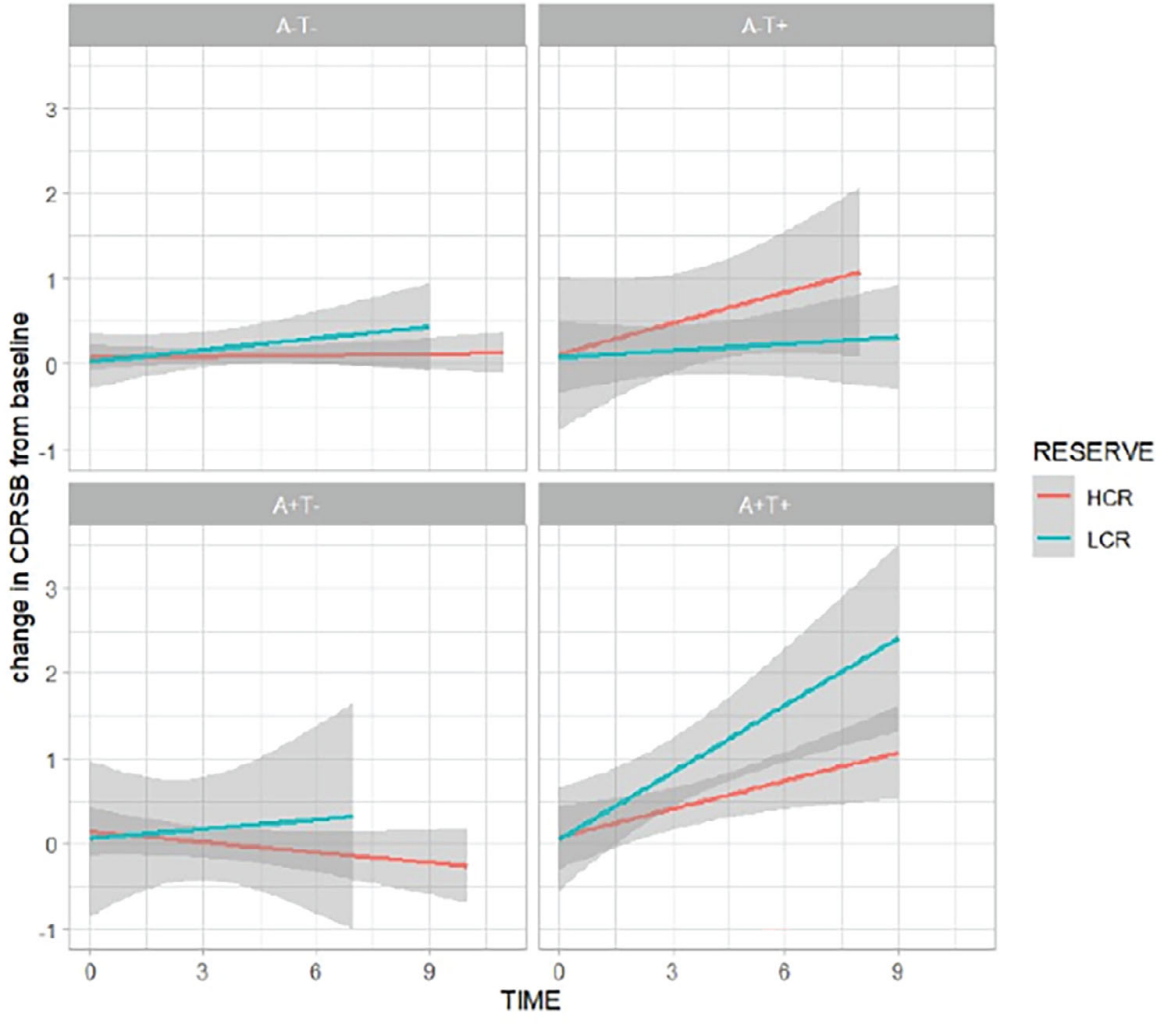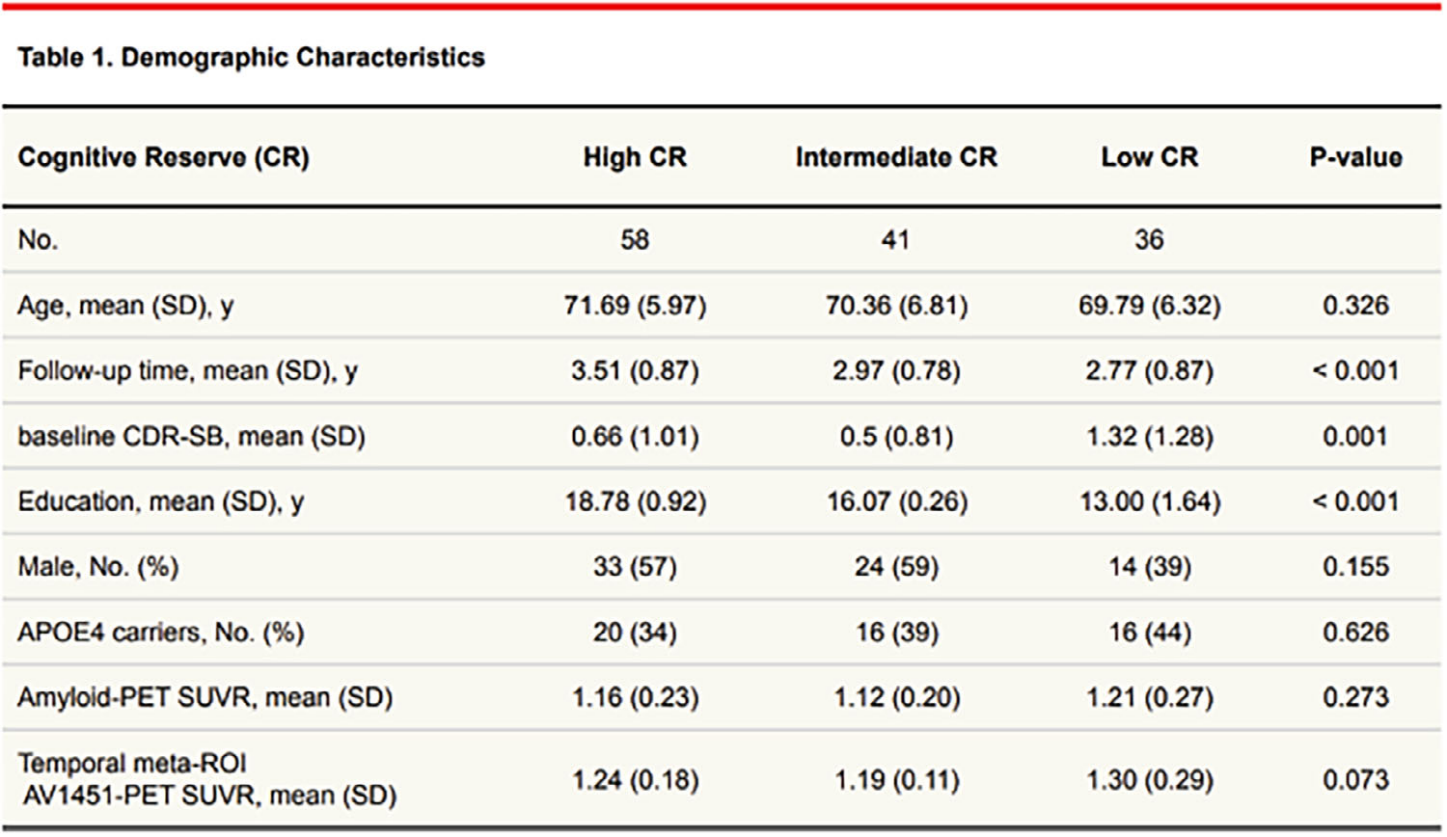# Association of amyloid and tau pathology in longitudinal cognitive reserve

**DOI:** 10.1002/alz.092182

**Published:** 2025-01-09

**Authors:** Wyllians Vendramini Borelli, Thomas Hugentobler Schlickmann, João Pedro Ferrari‐Souza, Marco Antônio de Bastiani, Andrei Bieger, Lucas Porcello Schilling, Pedro Rosa‐Neto, Eduardo R. Zimmer

**Affiliations:** ^1^ Federal University of Rio Grande do Sul, Porto Alegre, Rio Grande do Sul Brazil; ^2^ Memory Center, Hospital Moinhos de Vento, Porto Alegre, RS Brazil; ^3^ Universidade Federal do Rio Grande do Sul, Porto Alegre, Rio Grande do Sul Brazil; ^4^ Brain Institute of Rio Grande do Sul, PUCRS, Porto Alegre, RS Brazil; ^5^ Translational Neuroimaging Laboratory, The McGill University Research Centre for Studies in Aging, Montréal, QC Canada; ^6^ Brain Institute of Rio Grande do Sul ‐ Pontifícia Universidade Católica do Rio Grande do Sul, Porto Alegre, Rio Grande do Sul Brazil

## Abstract

**Background:**

Higher educational attainment is frequently associated with lower risk of cognitive decline. Many neuroprotective factors have been suggested as the biological underpinnings of cognitive reserve (CR), though the neural basis of CR is yet unclear. Herein, we aim at investigating the effect of CR using educational attainment in individuals stratified by amyloid and tau status.

**Method:**

A total of 135 participants, classified as cognitively unimpaired (CU) or Mild Cognitively Impaired (MCI), were selected from the ADNI cohort. All individuals that presented longitudinal measures and available amyloid and tau PET at baseline were included in this study. Formal years of education were selected as a proxy of cognitive reserve in this study, and individuals were classified into high CR (>17 years of education), intermediate CR (between 17 and 16), and low CR (<16 years of education) based on interquartile range. This analysis was in accordance with the most recent research framework to identify the mechanisms of cognitive reserve. A linear mixed‐effect model was performed to evaluate the interaction of cognitive reserve in the relationship between cognitive decline and AT status. The model was adjusted for age, sex, baseline diagnosis. Data is presented in mean±SD.

**Result:**

A total of 58 high CR (71.7±5, 57% males), 41 intermediate CR (70.4±6, 59% males) and 36 low CR (69.8±6.4% males) were included (Table). At baseline, these individuals were divided as A‐T‐ (n=69), A‐T+ (n=11), A+T‐ (n=27) and A+T+ (n=28). Mean follow‐up time of the sample was 3.15±0.9 years. Linear mixed‐effect model showed a significant interaction of cognitive reserve in the relationship between CDR‐SOB and A+T+ status (beta = 0.24, p‐val = 0.019)**,** after adjusting for covariates. Sensitivity analysis adjusting for APOE status also demonstrated the same relationship.

**Conclusion:**

The neuroprotective effect of cognitive reserve was prominent in individuals with both amyloid and tau pathology, but not in the Alzheimer's pathological changes (A+T‐). Understanding the neural basis of cognitive reserve may greatly improve the advancements in therapeutic targets for secondary prevention of cognitive decline in individuals with identified Alzheimer's disease pathology.